# Role of Calcium Channels in Heavy Metal Toxicity

**DOI:** 10.1155/2013/184360

**Published:** 2013-01-30

**Authors:** Carla Marchetti

**Affiliations:** Istituto di Biofisica, Consiglio Nazionale delle Ricerche, 16149 Genova, Italy

## Abstract

The role of voltage-dependent Ca channels (VDCC) in the membrane permeation of two toxic metals, lead (Pb) and cadmium (Cd), was studied in mammalian cells. Both metals interact with Ca-binding sites, but, while Cd influx appears to occur mainly through the same pathways as Ca, Pb is also rapidly taken up by different passive transport systems. Furthermore, I compared the effect of Cd in two Chinese hamster ovary (CHO) cell lines, a wild-type and a modified cell line, which were permanently transfected with an L-type VDCC. When cultures were subjected to a brief (30–60 min) exposure to 50–100 **μ**M Cd, apoptotic features, metal accumulation, and death were comparable in both cell lines although, in transfected cells, the effect of Cd treatment was partially prevented by nimodipine (VDCC antagonist) and enhanced by BayK8644 (VDCC agonist). Thus, expression of L-type Ca channels is not sufficient to modify Cd accumulation and sensitivity to a toxicological significant extent and while both Cd and Pb can take advantage of VDCC to permeate the membrane, these transport proteins are not the only, and frequently not the most important, pathways of permeation.

## 1. Introduction

Metals are used by biological systems because of their catalytic versatility, and some of them, namely, sodium (Na), potassium (K), calcium (Ca), and magnesium (Mg) are among the essential nutrients of living cells. In contrast, other metals can be fatal to mammalian cells even in tiny amounts. A common classification tends to sort these potentially toxic metals in to two classes: those required by living organisms as essential micronutrients and those devoid of any biological function and thus potentially toxic even at very low concentration, such as cadmium (Cd), chromium (Cr), mercury (Hg), and lead (Pb). The difference between toxic and nontoxic metals is hard to define. Even micronutrients, such as cobalt (Co), copper (Cu), iron (Fe), manganese (Mn), molybdenum (Mo), and zinc (Zn) can be detrimental to living organisms, when present in excessive levels, and a refined equilibrium between deficient and toxic concentrations has to be maintained. This is particularly important for very specialized tissues, such as the brain, where metals induce oxidative damage and some of the essential micronutrients, such as Fe, Zn, and Cu, have been implicated in etiology and development of different neurological and neurodegenerative diseases [1, 2]. Less obviously, living organism may find use for nonessential toxic metals in extreme condition. An elegant example of unexpected biological function of Cd has been recently reported in marine diatoms [3].

Because most toxic metals have entered the environment as a consequence of historical handcraft and industrial processing, specialized cells might not have developed the intrinsic ability to distinguish between physiological and toxic elements. For example, Cd and Pb represent a threat for mammalian cells, because they are able to replace or *mimic* essential metals in the first or early step of transport and metabolism, but then are incapable of mediating subsequent vital functions [4, 5]. The toxic effects are then a consequence of both impairment of transport systems and accumulation into the cell. Recent research has gathered an increasing body of evidence for the interaction of toxic metals with intracellular components that lead to cellular injury and defense, but the mechanisms of transport of these metals and metal-containing species across plasma membranes remain to be fully characterized. 

Cd and Pb mimic Ca and Zn at their specific sites and bind calmodulin [6, 7], protein kinase C [8–10], and synaptic proteins [11]. These metal ions antagonize Ca influx through voltage-dependent and receptor-operated Ca channels, and these channels are regarded as the major route of their entry into mammalian cells. 

Both Cd and Pb are well-known specific blockers of voltage-dependent Ca channels (VDCC), but the mechanism of block is quite different. While Cd binds to the high affinity site inside the VDCC pore [12, 13], Pb competes with Ca at a site external to the VDCC, and the binding is not voltage-dependent [14]. Given these differences in the mechanism of interaction, permeation of these metals through VDCC is also different. VDCCs mediate Cd influx in excitable cells [15–18], including mammalian neurons [19], and have been proposed to participate in Cd uptake also in cells from nonexcitable tissues [20]. In a previous work of my laboratory, we showed that in certain cells Cd permeation occurs mainly through VDCC of the L-type [19, 21]. However, in other studies, the presence of VDCC *per se* did not seem to enhance sensitivity to Cd; for example, VDCCs expressing (PC12) and nonexpressing (PC18) cells showed comparable LD_50_ for Cd [18], and the role of this pathway in the induction of cell death appears questionable.

The situation is even more complicated for Pb, whose chemical basis for mimicking Ca is not obvious [4] and which is known to adapt to structurally diverse binding geometries [22]. In cerebellar granule neurons, Pb uses at least three pathways of permeation and besides voltage-dependent calcium channels (VDCC) and NMDA-activated channels, is rapidly taken up through passive transport system [23, 24].

In this paper I will present experimental evidence concerning transport of Pb and Cd across cellular membranes and the relative contribution of VDCC in their uptake.

## 2. Materials and Methods

### 2.1. Cell Culture

Cerebellar granule neurons were prepared from 8-day-old Wistar rats as previously described [19, 25], plated on 20 mm poly-L-lysine coated glass coverslips and maintained in basal Eagle's culture medium, supplemented with 10% fetal calf serum, 100 *μ*g/mL gentamicin and 25 mM KCl, in a humidified 95% air/CO_2_ atmosphere at 37°C. Cultures were treated with 10 *μ*M cytosine arabinoside from day 1 to minimize the proliferation of nonneuronal cells. Experiments were carried out in cultures between 5 and 13 days *in vitro*.

Wild-type Chinese hamster ovary (CHO) cells were obtained from American Type Culture Collection. CHO stably transfected with cDNA encoding for several subunits of voltage-dependent calcium channels (VDCC) were a generous gift of Franz Hofmann and Norbert Klugbauer (Institut für Pharmakologie und Toxikologie, Münich, Germany). The cells used in this study are termed CHOC*α*9*β*_3_ *α*_2_/*δ*4 (abbreviated CHOC*α*) to indicate that they express the *α*_1C-b_ subunit of VDCC, as well as *β*_3_ and *α*_2_/*δ*4 subunits from smooth muscle [26].

Both wild-type CHO and CHOC*α* cells were maintained in DMEM medium (Sigma Chemical Co, St. Louis, MO, USA) supplemented with 10% bovine serum, in a 5% CO_2_ humidified atmosphere at 37°C. For CHOC*α* cells, the culture medium was routinely supplemented with antibiotic geneticin G418 20 *μ*gr/mL. For the experiments, cells were plated in 12-multiwell trays at a density of 2 × 10^5^ cells/mL.

### 2.2. Microscopy

Measurement of intracellular Cd and Pb was performed on monolayer cultures plated on glass coverslips. For photometric measurements, cells were incubated with 6 *μ*M of the cell-permeant Fura2-AM ester form of the dye for 45 min at 37°C and then washed several times with standard saline at room temperature, as previously described [19, 23]. Cd and Pb were applied in the absence of Ca and treatment was terminated by the addition of the membrane-permeant metal chelator (N,N,N′,N′-tetrakis-2-pyridyl methyl ethylenediamine, TPEN, 100 *μ*m).

For confocal microscopy, cells were incubated with Oregon Green 488 Bapta-1, a fluorescent indicator, whose fluorescence intensity is increased upon binding Ca, Cd, or Pb. Cells were imaged using a confocal laser scanning microscope Nikon PCM2000 (Nikon Instr., Florence, Italy) with an oil immersion 100 × objective  (Na = 1.3), coupled to a 50 *μ*m confocal pinhole condition [27].

### 2.3. Cd Treatment

Cells were subjected to 30–60 min treatments with 50 or 100 *μ*M CdCl_2_ in culture medium without serum. When specified, the medium was supplemented with 30 mM KCl to depolarize the cell membrane and with the following dihydropyridines (DHPs) : BayK  8644, which is an L-type VDCC agonist, or nimodipine, a channel antagonist. Stock solutions of nimodipine and BayK (10 mM) were made up in 100% ethanol and diluted in medium to the final concentration. High KCl alone, as well as DHPs (no Cd added), had no effect on cell viability and appearance. After incubation cells were washed three times with PBS containing 1 mM EDTA, and fresh medium containing serum was replaced. Cultures were kept in the incubator for another 18–24 hours before testing. Cell viability in each sample was assessed by trypan-blue exclusion assay. All chemicals, culture media and sera, were from Sigma-Aldrich Italy.

### 2.4. Capacitance and Current Measurements by Patch-Clamp Technique

Membrane currents were measured from wild-type CHO and permanently transfected CHOC*α* cells in whole-cell clamp configuration by a patch-clamp Axopatch amplifier (Molecular Devices Corporation, Union City, CA, USA). Electrodes were manufactured from borosilicate glass capillaries (Hilgenberg GmbH, Malsfeld, Germany) and had resistance of ≈4 M*Ω*. Voltage stimulation and data acquisition were performed by a PC through a Digidata 1440A interface and Pclamp-10 software (Molecular Devices). Currents were low-pass filtered at 5 kHz and digitized at 10 kHz. Capacitance transients were minimized by analog compensation, and the value obtained by this compensation was taken as an estimate of the cell capacitance. All currents traces were further corrected for leak and residual transients by a computer generated P/4 protocol. The holding potential was −80 mV in all the experiments. Current traces were analyzed with Clampfit-10 and Sigma Plot (Jandel Scientific, Erkrath, Germany) software. 

Cells were continuously superfused by gravity flow (10 mL/min) with a solution containing (in mM) NaCl 140, KCl 5.4, CaCl_2_ 1.8, Hepes 5. The pH was 7.4. The internal (pipette) solution contained (in mM): CsCl 20, CsOH 110, Aspartic acid 100, EGTA 5, Hepes 5, with pH adjusted to 7.3 with Trizma base. Calcium currents were recorded in similar external solution containing 130 mM NaCl and 5 mM CaCl_2_. Wild-type CHO cells do not possess prominent voltage-dependent currents, and CHOC*α* only contained the L-type VDCC under study; therefore there was no need to antagonize the current through other voltage-dependent channels to resolve Ca currents. The application of modifiers, such as agonist and antagonist DHPs, was accomplished by gravity flow; control ion substitution experiments showed that the external bath was completely changed in 10 sec, which was the maximal stimulation rate in these experiments.

### 2.5. Absorption Spectroscopy Cd Determination

Cells were exposed to Cd in culture medium without added serum. After treatment, cultures were washed three times with PBS containing 1 mM EDTA and harvested immediately. Viable cells were counted in triplicate in each sample. Cells were then washed again three times with the above buffer, resuspended in distilled water and disrupted by sonication (Sonopuls Ultrasonic Homogenizer, Bandelin) for 2 minutes in an ice bath. The total Cd content of each sample was measured by Flameless Atomic Absorption Spectroscopy (FAAS) using a Perkin Elmer Spectrophotometer (Model 1100 B) equipped with a graphite furnace (Model HGA 700). The Cd content was normalized to the volume occupied by the cells and molarity was calculated (see results). 

### 2.6. Statistical Analysis

Data are presented as mean ± standard error of the mean in at least 3 experiments. Statistical significance was evaluated by Student-Newman-Keuls multiple comparison test (In Stat, GraphPad Software). 

## 3. Results And Discussion

### 3.1. Influx of Toxic Metals into Mammalian Cells

Influx of toxic divalent metal ions into mammalian cells can be monitored in cells preloaded with specific fluorescent probes, Fura2 [17, 19, 23], or Oregon green. These fluorescent indicators were developed and are predominantly used for determining intracellular calcium levels, but they have relatively high affinity also for other polyvalent ions, including Cd and Pb. 

**Figure 1:**
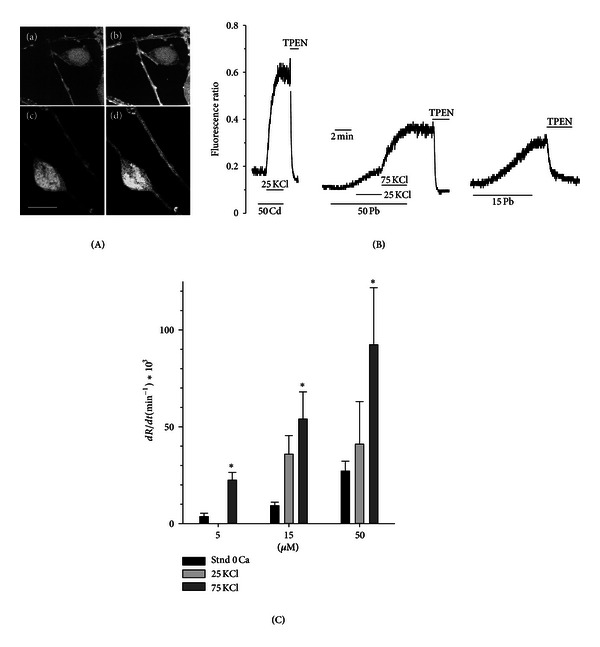
Uptake of Cd and Pb in cerebellar granule neurons measured by fluorescent dyes. (A) Confocal microscopy images of Cd (a, b) and Pb (c, d) uptake by cerebellar granule cells preloaded with the divalent metal-sensitive dye Oregon Green. In (a) and (c), neurons were bathed in a physiological saline. In (b) they have been superfused with a solution containing 0 Ca, 30 mM KCl and 100 *μ*M Cd Cl_2_, which caused the dye fluorescence to increase significantly. In (d), external solution contained 0 Ca and Pb 15 *μ*M, which permeates through the neuron membrane even in the absence of a depolarizing stimulus and also caused an increase in the dye fluorescence. Bar = 10 *μ*M. (B) Real-time recording of the influx of Cd and Pb in Fura-2 loaded cerebellar granule neuron and the effect of membrane depolarization. Cells were treated with the metals in nominal Ca-free solution. From left to right: time course of the fluorescence ratio, *R* = E340/E380, following application of 50 *μ*M Cd and membrane depolarization (25 mM KCl) and application of 15–50 *μ*M Pb in resting conditions and increasing depolarizations (25 and 75 mM KCl). (C) Summary of the results obtained in basal and in depolarizing solutions (25 and 75 mM KCl) with different doses of Pb. The time course of *R*(t) was approximated by a straight line and the slope dR/dt was calculated in each case, as a measure of Pb influx. Data are mean ±  SEM in 4 experiments and *indicates significantly different from basal (no depolarization) for each dose of Pb (*P* < 0.05).

Figure 1 shows how exposure to a solution containing a toxicologically significant concentration of Cd or Pb causes a sizeable increase in Oregon Green fluorescence, similar to that seen following Ca influx [28]. This demonstrates feasibility of this probe as toxic metal influx indicator. In this experiment, while Cd uptake was triggered by depolarization, indicating the essential contribution of VDCC (Figure 1(A) (a)-(b)), neurons rapidly accumulate Pb even in the absence of a specific stimulus (Figure 1(A) (c)-(d)).

Figure 1(B) shows real-time measurements of Cd and Pb influx in Fura2-loaded neurons [19, 23]. The influx was quantified by measuring the fluorescence emission ratio *R* (E_380_/E_340_), and membranes were depolarized by increasing concentration of external KCl [25]. In the left panel, the fluorescent ratio, E_340_/E_380_, increased negligibly when 50 *μ*M Cd was applied in resting condition, but the rate of rise, dR/dt, was enhanced by >60 times following depolarization with 25 KCl. In contrast, Pb (15–50 *μ*M) determined a sizeable increase of *R* even in the absence of any specific stimulus that opens VDCC, and dR/dt was increased by 5 times from the resting level with 75 mM KCl (Figure 1(C)). Fast uptake of Pb in the absence of any specific stimulus appears to be a peculiar feature of neurons and may be linked to the specific lipidic composition of the neuronal membranes, while the role of VDCC is likely to significant, but not essential [23].

### 3.2. Role of VDCC in Cd-Induced Mortality

**Figure 2:**
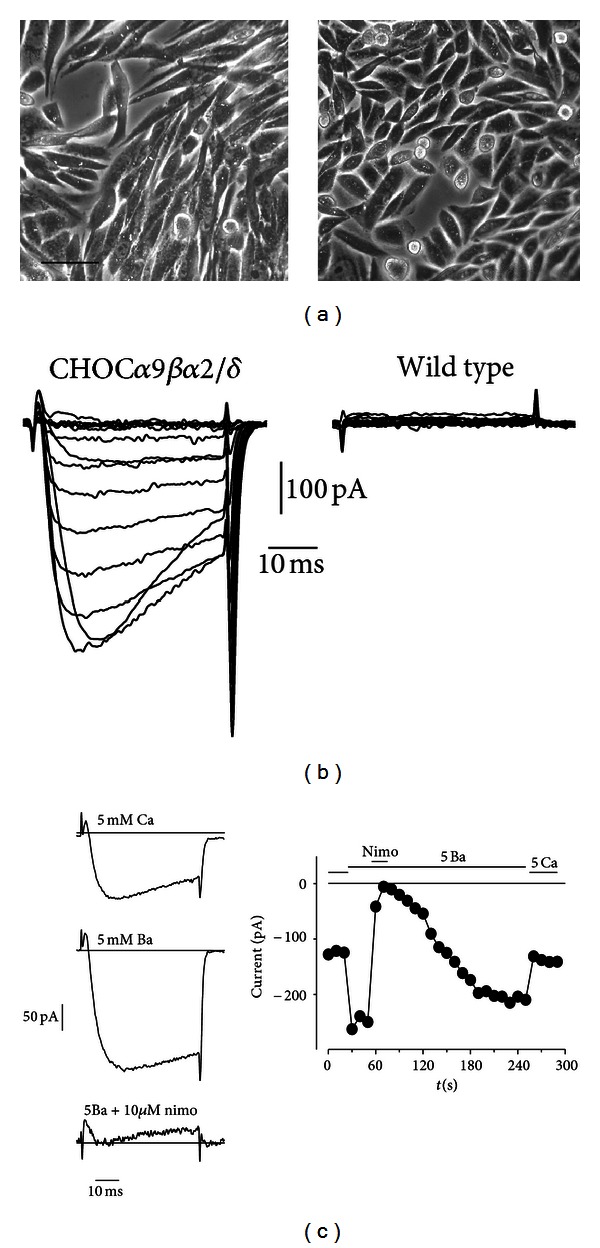
CHOC*α* cells express L-type VDCC. (a) Representative microphotographs of monolayer cell cultures of permanently transfected CHOC*α* cells (left) and wild-type CHO cells (right). Bar = 35 *μ*M. (b) Representative current traces evoked by depolarizing voltage steps of 40 ms duration from −60 to +80 mV from a holding potential of −80 mV in a CHOC*α* cell (left) and a wild-type CHO cell (right). The external solution contained 5 mM CaCl_2_. This protocol evoked voltage-dependent calcium current in the permanently transfected cell, while no current was present in CHO cell of the wild-type. (c) Characterization of the Ca current in CHOC*α* cell. The current was increased by more than 50% when the external solution was changed from 5 mM CaCl_2_ to 5 mM BaCl_2_, and it was reversibly blocked by 10 *μ*M nimodipine. Current traces evoked by 50 ms depolarizing steps from −80 mV (holding potential) to +10 mV in the three conditions are shown on the left. The graph on the right shows the time course of the experiment.

As VDCC channels, and in particular those of L-type [15, 19] play a prominent role in uptake of Cd by excitable cells, the involvement of these channels in Cd toxicity was investigated further. Tests of mortality, chromatin condensation, and DNA fragmentation showed that a pulsed Cd treatment induces delayed apoptotic cell death in VDCC-containing insulinoma cells and that nimodipine protects against Cd-induced apoptosis and necrosis in these cells, supporting a specific involvement of L-type VDCC [21]. The same treatments were largely harmless in VDCC-free HeLa cell cultures, in which neither death nor DNA fragmentation was observed. Therefore, excitable cells appear more susceptible than nonexcitable epithelial-like cells to Cd accumulation. However, it is not clear whether the mere presence of VDCC can make cells more vulnerable to toxic metal injury. To answer this question we have compared the effects of Cd in two cell types that are virtually identical, except for the expression of VDCC in one of the two. This approach is made possible by the availability of a CHO cell line that has been permanently transfected with VDCC *α*_1_, *β*, and *α*_2_/*δ* subunits, CHOC*α*9*β*_3_ *α*_2_/*δ*_4_ cells [26, 29]. Prominent voltage-dependent Ca currents were recorded from the permanently transfected cell line, while such currents are completely absent in wild-type CHO (Figure 2(a)). The currents are larger with Ba as charge carrier and are sensitive to DHP modulation (Figure 2(b)), as expected for an L-type Ca channel.

**Figure 3:**
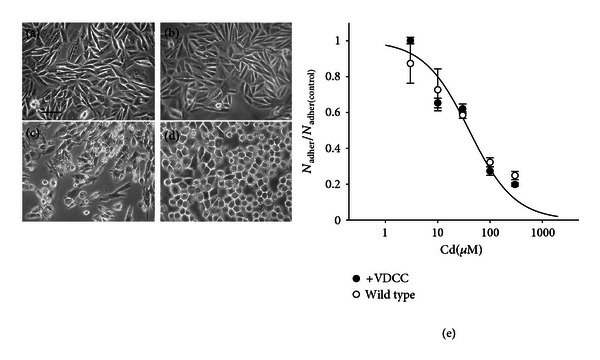
Effect of pulse treatment with Cd in CHOC*α* VDCC-expressing and wild-type CHO cells. Microphotographs of control (a, b) and Cd-treated (c, d) cells: (a, c) VDCC-expressing CHOC*α* cells and (b, d) wild-type CHO cells. Both cell types were incubated in 100 *μ*M Cd for 60 minutes in the presence of 30 mM KCl. Pictures were taken 24 h after wash of the metal. Bar = 35 *μ*M. The graph (e) shows the effect of a 30 min pulse treatment with Cd on cell adhesion, as a function of concentration. Trypan-blue excluding adherent cells were counted 24 h after wash of the metal. Points are average ±  sem of 3 experiments in the same condition in CHOC*α* (filled circles) and wild-type CHO (empty circles) and were best fitted to the function. N_adher_/N_adher(control)_ = 1/(1 + ([Cd]/ED_50_)), where N_adher_/N_adher(control)_ is the number of adherent cells after Cd treatment normalized to the number of adherent cells in control culture; [Cd] is the concentration of Cd and ED_50_ is the concentration of Cd that causes detachment from the substrate of 50% of cells. The best fit yielded ED_50_ = 40 *μ*M for CHOC*α* and 43 *μ*M for wild-type CHO. The two curves are overlapped. In contrast with the different appearance, the two cell types were similarly affected by Cd treatment.

Both cell types (wild-type and CHOC*α*) were subjected to a “pulse treatment” with 50 or 100 *μ*M Cd in serum-free culture medium and observed after 18–24 hour after wash. Cell suffering and mortality was negligible immediately after the treatment, but it was evident after at least 16 hours. As previously observed [21, 30, 31], Cd accumulates into the cell during treatment, but the toxic mechanism leading to cell death becomes effective at a later time.  Figure 3 shows the appearance of the cell monolayer 24 hours after an incubation of 30 min with 100 *μ*M Cd. Cells exposed to Cd were of smaller size than control cells and had an increased tendency to detach from their neighbors and from the substrate and float. In addition, CHOC*α* VDCC-expressing cells frequently acquired an irregular shape, with shredded edges (Figure 3). The number of viable cells was estimated from the number of adherent, trypan-blue excluding cells, and this number was normalized to that of adherent viable cells in control conditions, as in our previous works [21]. Despite some morphological features, the dose dependence of Cd sensitivity was very similar in the two cell types (Figure 3). Therefore, by this approach it is not possible to demonstrate a clear role of VDCC in mediating Cd-induced cell detachment.

**Figure 4:**
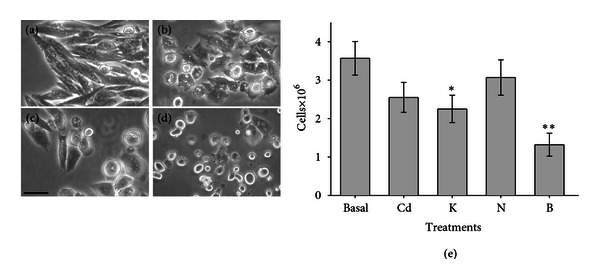
Effect of dihydropyridines on Cd cytotoxicity in VDCC-expressing CHOC*α* cells. Cells were treated for 30 min in (a) control medium containing 30 mM KCl, (b) 100 *μ*M  Cd + 30 mM  KCl, (c) 100 *μ*M  Cd + 30 mM  KCl + 1 *μ*M nimodipine, (d) 100 *μ*M  Cd + 30 mM  KCl + 1 *μ*M  BayK8644. Note the partial recovery in shape of the cells treated in the presence of nimodipine and definitive lost of adhesion when the treatment was performed in the presence of BayK. Bar = 25 *μ*M. The graph (e) represents counts of viable adherent cells in the same experiment (average ±  sem in 3 samples) in control (basal), following for a 30 min treatment with 100 *μ*M Cd (Cd), Cd + 30 mM  KCl (K), Cd + 30 mM  KCl and 1 *μ*M nimodipine (N), and Cd + 30 mM  KCl and 1 *μ*M BayK (B). * indicates significantly different from control with *P* < 0.05, and ** with *P* < 0.0001.

In subsequent experiments, we challenged the cells with Cd in the presence of 1 *μ*M DHP modifiers. When VDCC-expressing CHOC*α* cells were exposed to Cd in the presence of the antagonist (nimodipina) or agonist (BayK 8644), the effect of the metal was clearly modified with respect to cells exposed to Cd alone (Figure 4), with a sizeable increase of mortality in the presence of BayK and protection in the presence of nimodipine. DHP modifiers were largely ineffective in wild-type CHO cells exposed to Cd (not shown). Therefore, it seems that the actual contribution of L-type VDCC to Cd uptake is clearly underscored only in the presence of these modifiers.

**Figure 5:**
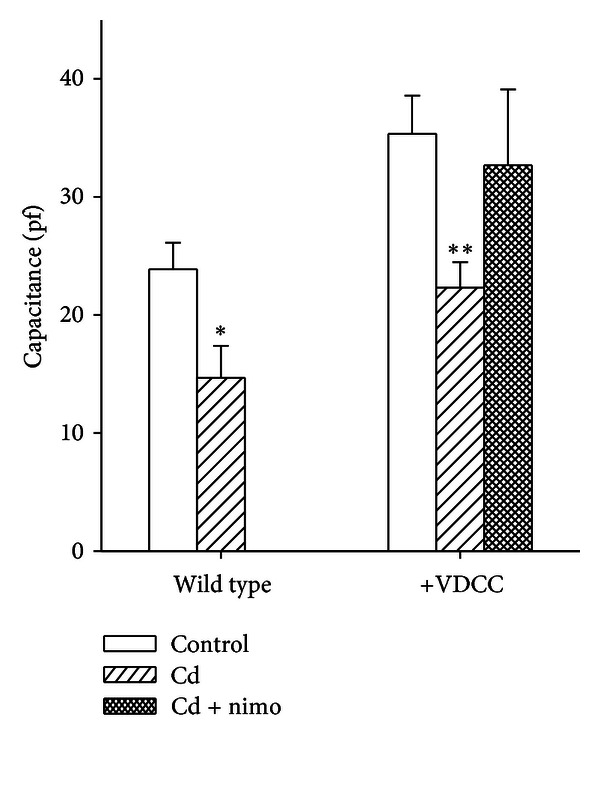
Evidence of Cd-induced apoptosis: change in cell capacitance. Cells were incubated with 100 *μ*M Cd for 60 minutes, and electrical measurements were performed 24 h after wash. Membrane capacitance was estimated from transient compensation (see Section 2.4). Bars represent mean ±  sem. Treatment with Cd caused shrinkage of all cell, as revealed by reduction of the cell capacitance in both wild-type (*n* = 15 in control and *n* = 6 with Cd treatment) and CHOC*α* cells (*n* = 17 in control and *n* = 39 with Cd treatment). Nimodipine protected CHOC*α* cells from Cd-induced shrinkage (*n* = 10). * indicates significantly different from control with *P* < 0.05, and ** with *P* < 0.001.

Another indicator of apoptosis is cell shrinkage, which can be quantified by cell capacitance measurements. Both wild-type and CHOC*α* cells showed a decrease in cell capacitance 18–24 hours after Cd treatment. In wild-type, cell capacitance was 24 ± 2 pF  (*n* = 15) in control and 15 ± 3 pF (*n* = 6) after incubation with 100 *μ*M Cd, significantly different from control with *P* < 0.05. In CHOC*α* cells, cell capacitance was 35 ± 3 pF (*n* = 17) in control and 22 ± 2 pF (*n* = 39) after incubation with 100 *μ*M Cd, significantly different from control with *P* < 0.001. In CHOC*α* cells, the effect of Cd treatment was partially prevented by nimodipine: the capacitance was 35 ± 3 pF (*n* = 10) and the difference between this condition and control condition was not significant (Figure 5). This indicates again that the contribution of L-type VDCC in Cd-induced toxicity is evident only in the presence of DHPs. 

**Figure 6:**
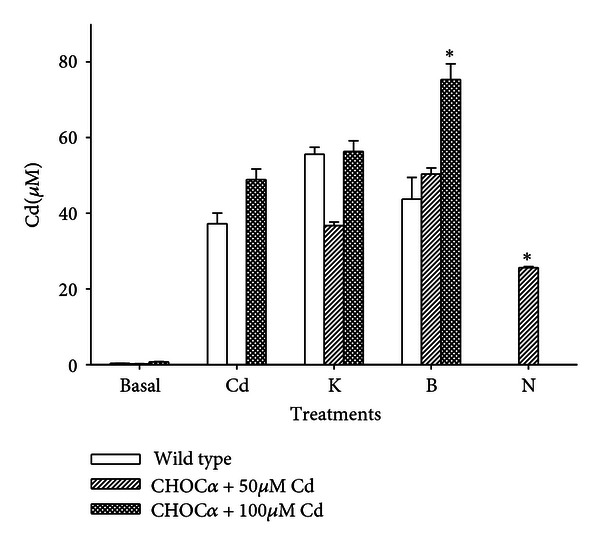
Total Cd accumulation measured by FAAS in CHOC*α* and wild-type CHO cells. Both cell types were incubated for 1 hour in medium containing 0, 50, or 100 *μ*M Cd and other modifiers. Cd determination was normalized to the cell volume (see text). Treatments were as follows: control 0 Cd (basal), 60 min Cd (Cd), Cd + 30 mM  KCl (K), Cd + 30 mM  KCl, and 1 *μ*M BayK (B), Cd + 30 mM  KCl and 1 *μ*M nimodipine (N). Depolarization (treatment K) did not increase significantly the intracellular concentration of Cd (*P* > 0.05). In CHOC*α* cells, but not in wild-type CHO, BayK significantly enhanced and nimodipine significantly reduced Cd accumulation, both with *P* < 0.001 (*), with respect to the same treatments in depolarization. Bars represent mean ±  sem in at least 10 experiments in each group.

Finally, we performed measurements of total Cd accumulation during 60 min incubation time by atomic absorption spectroscopy. For these experiments, cells were harvested immediately after the treatment, and the total amount of Cd was normalized to the cell volume, estimated from the single-cell capacitance. Assuming that both cell types are approximately spherical and have a specific membrane capacitance of 1 *μ*F/cm^2^, 10^6^ wild-type CHO cells comprise a volume of 11 *μ*L, while 10^6^ CHOC*α* cells a volume of 19.5 *μ*L. This difference is not negligible, and with this method, the results were considerably more reproducible than using a standard normalization to the total protein content. Data shown in Figure 6 indicate that the difference in Cd accumulation is barely or not significant when both cell types are incubated in 100 *μ*M Cd with or without elevated KCl. On the other hand, BayK 8644 significantly enhanced and nimodipine significantly decreased metal accumulation in depolarized CHOC*α* cells treated with 50 or 100 *μ*M Cd, but not in wild-type CHO cells.

Toxicity of both Cd and Pb is mainly due to their ability to permeate not simply mammalian cell membranes, but specifically tight membrane barriers, such as the epithelial lining in the gastrointestinal tract, luminal membrane of proximal tubules in the kidney, and the blood-brain barrier. In early work, the influx of Pb and Cd into mammalian cells was frequently ascribed to permeation through calcium channels, and in particular L-type DHP-sensitive VDCC [16, 18, 20, 32]. More recently other types of VDCC, different from the classical DHP-sensitive L-type, have been implicated both in Cd [19, 33] and Pb [23] uptake. Moreover, Pb has been shown to use different pathways in several systems [34–37], and it is largely independent of VDCC even in neurons [23, 24]. 

Here I have shown that expression of L-type VDCC is not sufficient to modify Cd uptake to a toxicological significant extent, at least in the case of a brief, intense exposure, as those used in this study. Cd accumulation and sensitivity were comparable in two cell lines whose only difference was the presence of L-type VDCC in one of the two. Indeed, wild-type CHO cells, which do not possess VDCC, are vulnerable to Cd poisoning and take up Cd during relatively brief exposures to an extent sufficient to trigger cell death by apoptosis or necrosis similarly to VDCC expressing CHOC*α* cells. Although VDCCs play an important role in Cd uptake in CHOC*α* cells, as indicated by the sizeable effect of L-type channel DHP modifiers, wild-type CHO cells use other transport proteins with similar efficiency and outcome. 

It is thus clear that VDCCs are not the main mechanism responsible for toxic metal permeation through cell membranes and tight barriers. Rapid passive transport of Pb, independent of VDCC, was reported at the brain endothelium [34, 36, 37], and even protein-independent transport of lead has been described [35]. In astroglia cell models, the uptake of Pb was shown to be driven by store-depletion-activated channels [38] and by two distinct pH-sensitive transport mechanisms [39]. VDCC-independent uptake of Pb by cerebellar granule neurons was inhibited by La [24], and this observation can provide some clue as for the mechanism of permeation. The involvement of a La-sensitive store-depletion activated channel, as proposed in other systems [36, 37, 40], is unlikely because Pb influx did not require drainage of the stores. Another possibility is that Pb^2+^ enters the neuron via reverse operation of an exchanger similar (or identical) to the Na–Ca exchanger, which is also very sensitive to La block [41] and whose role in Zn permeation was demonstrated in cortical neurons [42]. Also this possibility was discarded because Na–Ca exchanger does not play a significant role when the Na concentrations are close to the physiological value, as in Esposito et al. [24]. It is more probable that Pb is taken up through a La-sensitive ion carrier similar to the Zn transporter found in brain neurons [43], whose identity and features still wait full definition.

Involvement of different transport proteins in Cd transport has been described. At the epithelial lining of the gastrointestinal barrier, DMT1, the main transporter that absorbs Fe in the brush border membrane of the mammalian intestine has been shown to carry also different toxic metals [44–46]. The role of this transporter in Cd absorption is underscored by the fact that iron deficiency creates a significant risk for increased cadmium exposure by increasing gastrointestinal absorption from 5% to as much as 20%. In general, a low level of essential metals favors uptake of nonessential (toxic) elements that compete for their site, and this stresses the importance of evaluating metal balance, in contrast with metal concentration. In addition, other metal-specific transporters have been identified at the cDNA level [47] and studied in expression systems [48]. 

## 4. Conclusion

Ionic mimicry is a useful framework to study the mechanisms of metal toxicity. Uptake of toxic metals, such as Cd and Pb, by mammalian cells of different tissues occurs through many different pathways, of which VDCCs have frequently been regarded as prominent. Because of the abundance of VDCC, excitable cells may be more susceptible to accumulate Cd and Pb than nonexcitable cells. In this chapter I have shown that although Cd and Pb can permeate through VDCC, this is not the most relevant mechanism of passive uptake of Pb, while the mere presence of VDCC is not sufficient to make cells significantly more vulnerable to Cd injury. Other factors, including different transport systems and expression of detoxification proteins, may be more relevant in the accumulation process of these toxic metals.
